# Nanoporous water oxidation electrodes with a low loading of laser-deposited Ru/C exhibit enhanced corrosion stability

**DOI:** 10.3762/bjnano.10.15

**Published:** 2019-01-11

**Authors:** Sandra Haschke, Dmitrii Pankin, Vladimir Mikhailovskii, Maïssa K S Barr, Adriana Both-Engel, Alina Manshina, Julien Bachmann

**Affiliations:** 1 Friedrich-Alexander University Erlangen-Nürnberg, Department of Chemistry and Pharmacy, Chair of Chemistry of thin film materials, Egerlandstrasse 3a, 91058 Erlangen, Germany; 2 Saint-Petersburg State University, Center for Optical and Laser Materials Research, Uljanovskaya 5, 198504 St. Petersburg, Russia; 3 Saint-Petersburg State University, Interdisciplinary Resource Center for Nanotechnology, Uljanovskaya 1, 198504 St. Petersburg, Russia; 4 Saint-Petersburg State University, Institute of Chemistry, Universitetskii pr. 26, 198504 St. Petersburg, Russia

**Keywords:** electrochemistry, nanostructures, noble metals, ruthenium catalyst, water splitting

## Abstract

For the oxidation of water to dioxygen, oxide-covered ruthenium metal is known as the most efficient catalyst, however, with limited stability. Herein, we present a strategy for incorporating a Ru/C composite onto a novel nanoporous electrode surface with low noble metal loading and improved stability. The Ru/C is coated on the pore walls of anodic alumina templates in a one-step laser-induced deposition method from Ru_3_(CO)_12_ solutions. Scanning electron microscopy proves the presence of a continuous Ru/C layer along the inner pore walls. The amorphous material consists of metallic Ru incorporated in a carbonaceous C matrix as shown by X-ray diffraction combined with Raman and X-ray photoelectron spectroscopies. These porous electrodes reveal enhanced stability during water oxidation as compared to planar samples at pH 4. Finally, their electrocatalytic performance depends on the geometric parameters and is optimized with 13 μm pore length, which yields 2.6 mA cm^−2^, or 49 A g^−1^, at *η* = 0.20 V.

## Introduction

**Figure 1:**
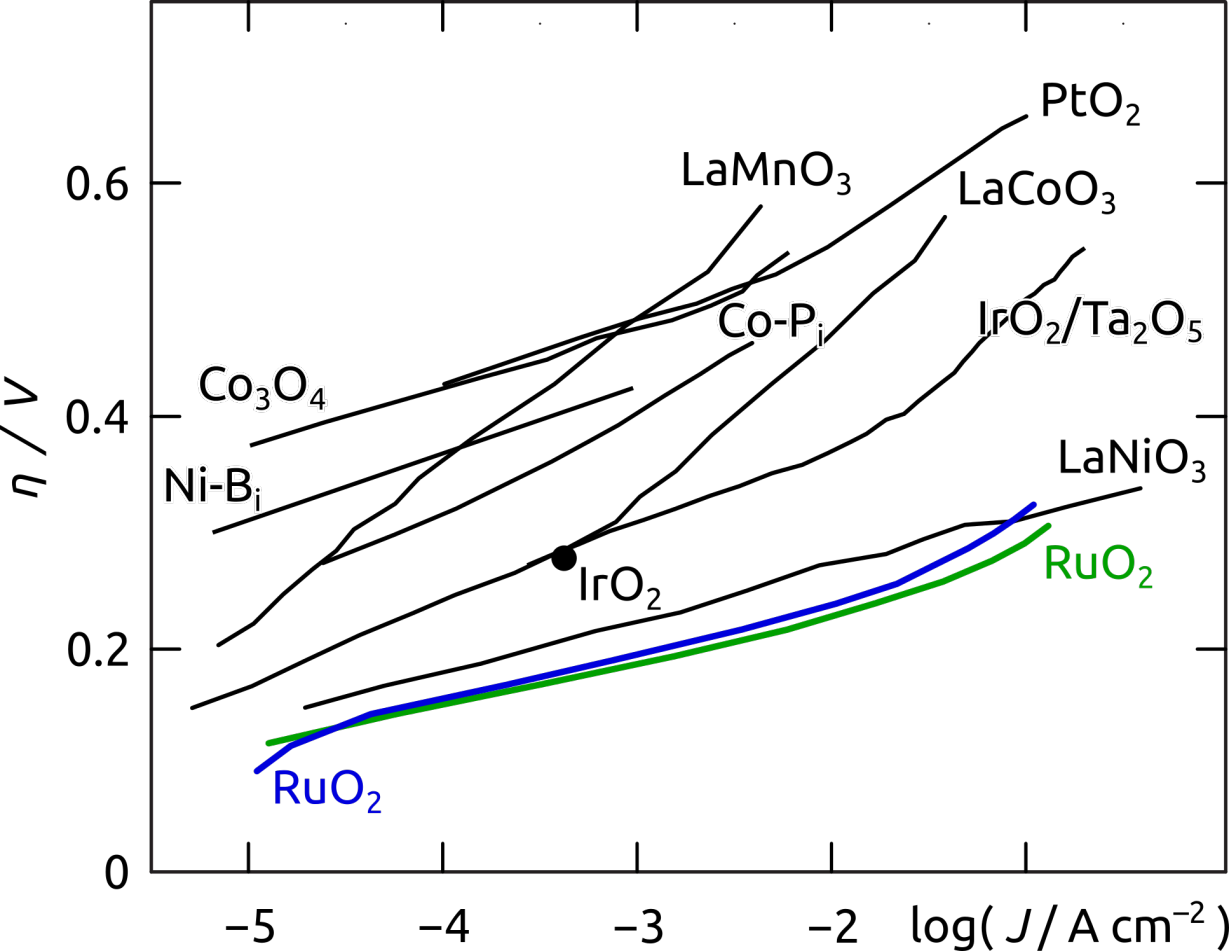
Selected oxygen evolution activities for planar state-of-the-art electrode materials (adapted from a review by Cook et al.) [9]. The overpotential *η* is shown here as a function of the logarithm of the current density *J*. The catalytic performance of ruthenium oxide in concentrated acidic and basic conditions is highlighted in green and blue, respectively.

The replacement of fossil fuels as the dominant global source of power by renewable energy sources has been and still is one of the major scientific and technological challenges faced by mankind. Among conceivable alternative energy sources, solar energy is the most suitable candidate due to its highest abundance on the global scale. Solar energy application on a large scale, however, necessitates its storage [1–4]. Here, nature provides the blueprint for the production of solar fuels by rearranging the chemical bonds of water to dihydrogen and dioxygen [1,5]. For the realization of artificial water splitting, catalysts are required for the rate-limiting half reaction, the dioxygen evolution, which must be driven at low overpotential (for maximizing conversion efficiency) [2]. The most active catalyst materials for this transformation are metallic iridium and ruthenium particles, the surface of which consists of the corresponding oxide (Figure 1) [6–7]. Of the two, ruthenium (as its ruthenium(IV) oxide at the surface) not only proves to be the more efficient catalyst but is also the more abundant, and thus, the more cost-effective material [8].

**Figure 2:**
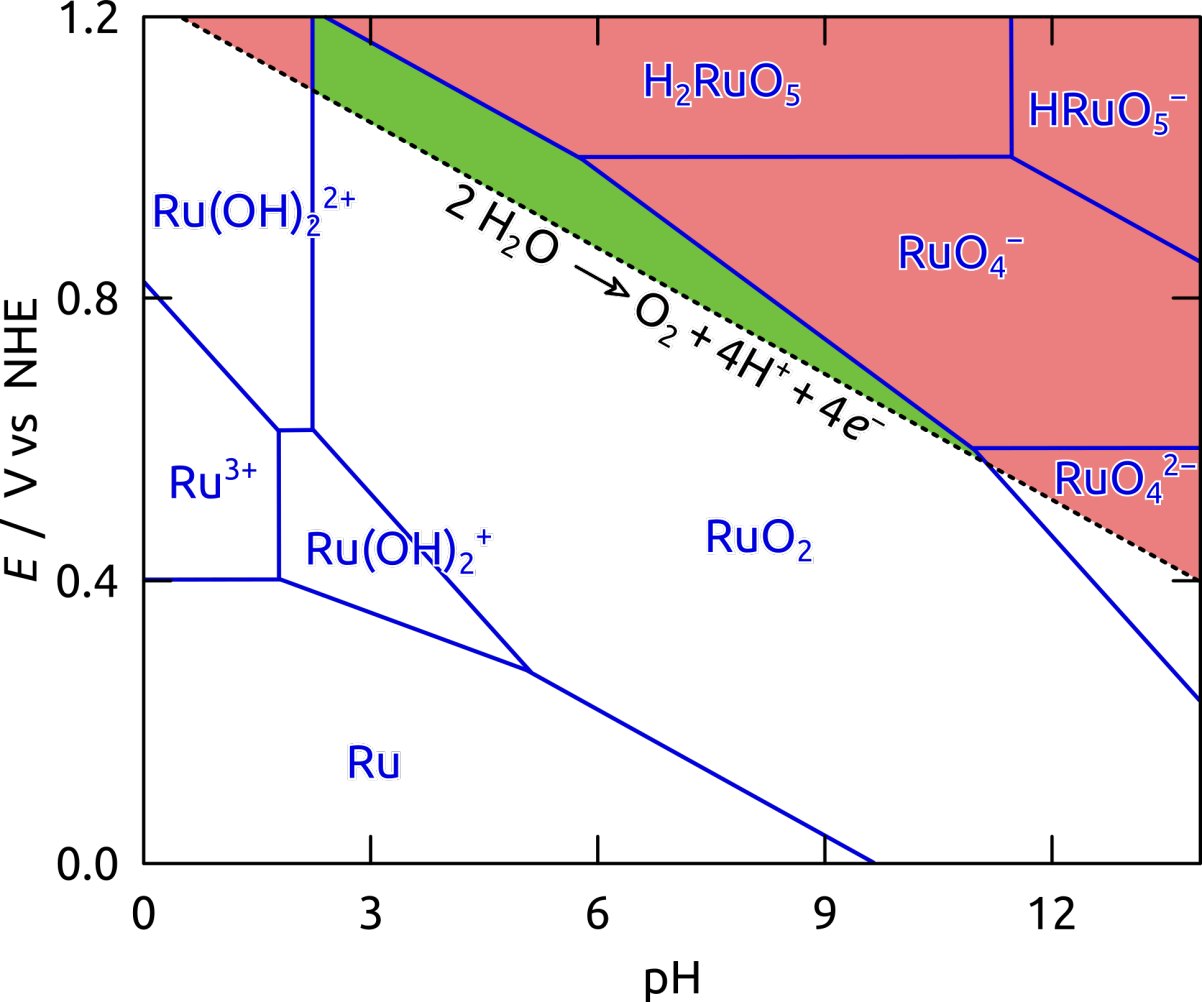
Pourbaix diagram for ruthenium in the presence of water (adapted from the Atlas of Eh-pH diagrams, Intercomparison of thermodynamic databases, Geological Survey of Japan Open File Report No.419) [12]. The limited thermodynamic stability of Ru is illustrated for positive potentials *E* in the range of 0 ≤ pH ≤ 14 (blue lines) with the corresponding pH-dependent equilibrium potential for the water oxidation reaction (black dotted line). Regions of predominant thermodynamic stability and dissolution of the solid in water oxidation conditions are color-coded in green and red, respectively.

However, its practical application is limited by its significant dissolution (corrosion) at high anodic potential over the whole pH range (Figure 2) [10–12]. One strategy to address this limitation has involved mixing metallic Ru (or its oxides) with other solids (such as Ir [13–18], Ta [19], or Pt [20], TiO_2_ [21], Ni and Co [22]). Another approach entails increasing the specific surface area, which allows one to generate current at lower overpotential, for example by supporting RuO_2_ nanoparticles on siliceous mesoporous materials [23–26], with mesoporous RuO_2_ [27], or with RuO_2_ supported on Sb-doped SnO_2_ nanoparticles [18,28]. A carbonaceous support has also been used, with the advantages of chemical durability and electrical conductivity, as demonstrated in the context of alcohol dehydrogenation [29–30]. Such a support, however, has not been applied to the water oxidation reaction so far.

In this paper, we address this goal with the synthesis of metal/carbon composites via the laser-induced deposition method already described for carbon-encapsulated Ag/Au nanoparticles (AgAu/C) [31–33]. This practically appealing one-step technique bases on the photo-induced decomposition of a dissolved organometallic complex and the subsequent self-organization into hybrid metal/carbon nanostructures with controlled composition and morphology [32]. With the appropriate choice of laser wavelength and solvent, which both need to be adjusted to the absorption behavior of the organometallic complex, metal/carbon composites (M/C) can be generated in a straightforward manner onto the surface of any substrate with 2D or 3D architecture [34].

We first establish a novel laser-induced deposition method for Ru/C on planar substrates from commercially available triruthenium dodecacarbonyl (Ru_3_(CO)_12_). We then transfer the successful deposition method to the functionalization of highly ordered nanostructured anodic alumina templates [35–39] (Note: as our system is based on pores of diameter >50 nm, it is macroporous according to the IUPAC definition; we will use the more general wording “nanoporous” and “nanotubular” in the rest of the paper). These novel metal/carbon nanostructures are characterized regarding their morphology and phase composition. Finally, the electrocatalytic water oxidation performance of planar and nanostructured Ru/C electrodes is studied at pH 4. The focus lies on (1) the optimization of the nanoporous geometry (variation of the pore length) towards obtaining reasonable current densities at low overpotential, (2) the minimization of corrosion via minimized overpotential and nanoparticle morphology, and (3) the minimization of noble metal loading. These efforts result in a very high activity (current per mass of noble metal) for electrocatalytic water oxidation.

## Results and Discussion

### Laser-induced deposition of planar hybrid Ru/C films

According to previous studies on the one-step laser-induced deposition method of AuAg/C composite, the choice of organometallic precursor, solvent, irradiation wavelength, and time crucially affect the quantity and quality of the coating [31,33,40]. For Ru/C deposition, we chose triruthenium dodecacarbonyl, Ru_3_(CO)_12_, as a 1 mg/mL 1,2-dichloroethane solution irradiated within its absorption band at 325 nm [41–42].

**Figure 3:**
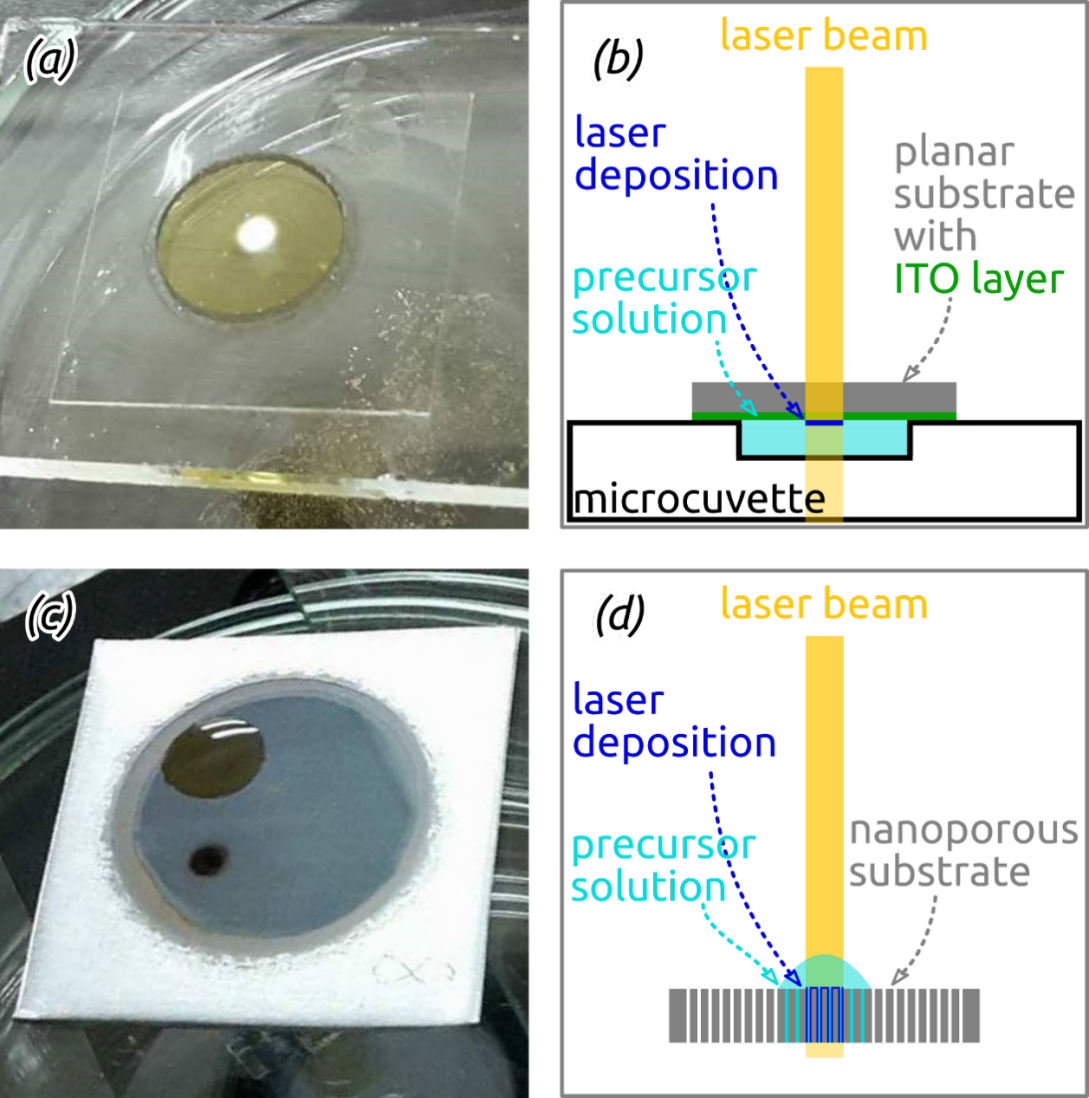
Photographs and schematic drawings of laser-induced chemical liquid deposition geometry on planar (a,b) and nanostructured samples (c,d) from Ru_3_(CO)_12_ solutions.

As planar substrates, microscope cover glasses provided with approx. 700 nm of heat-treated indium tin oxide (ITO, which serves as the electrical contact in subsequent electrochemical measurements) are placed for laser-induced coating on a microcuvette filled with the precursor solution. An unfocused He-Cd laser beam irradiates the substrate/solution interface from the substrate side for 30 min (Figure 3a,b).

**Figure 4:**
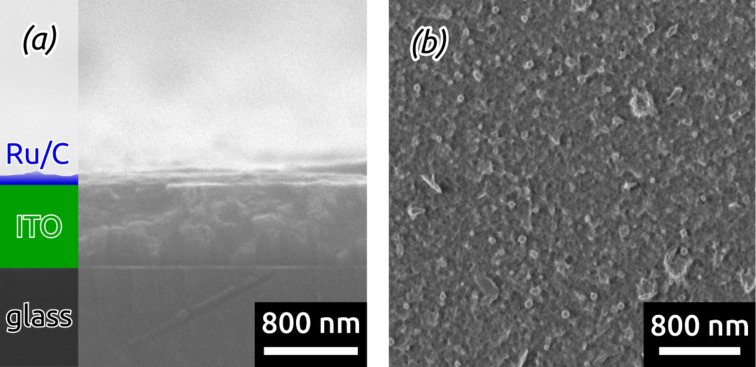
SEM images of planar samples coated with laser-induced Ru/C films in cross-section (a) and top-view (b). Laser-irradiation of Ru_3_(CO)_12_ solutions results in continuous and rough thin films deposited on ITO.

Scanning electron micrographs of a sample prove the successful deposition of a continuous albeit somewhat rough thin (≤20 nm) film (Figure 4a,b). Furthermore, energy-dispersive X-ray (EDX) analysis confirms the presence of Ru and C in the deposited film (see Figure S1 in Supporting Information File 1). This is the first demonstration of Ru/C hybrid material generated by laser-induced deposition. Furthermore, this represents the first use of a commercially available precursor in this method, which simplifies the procedure and renders it widely available.

### Laser-induced deposition on nanoporous substrates

**Figure 5:**
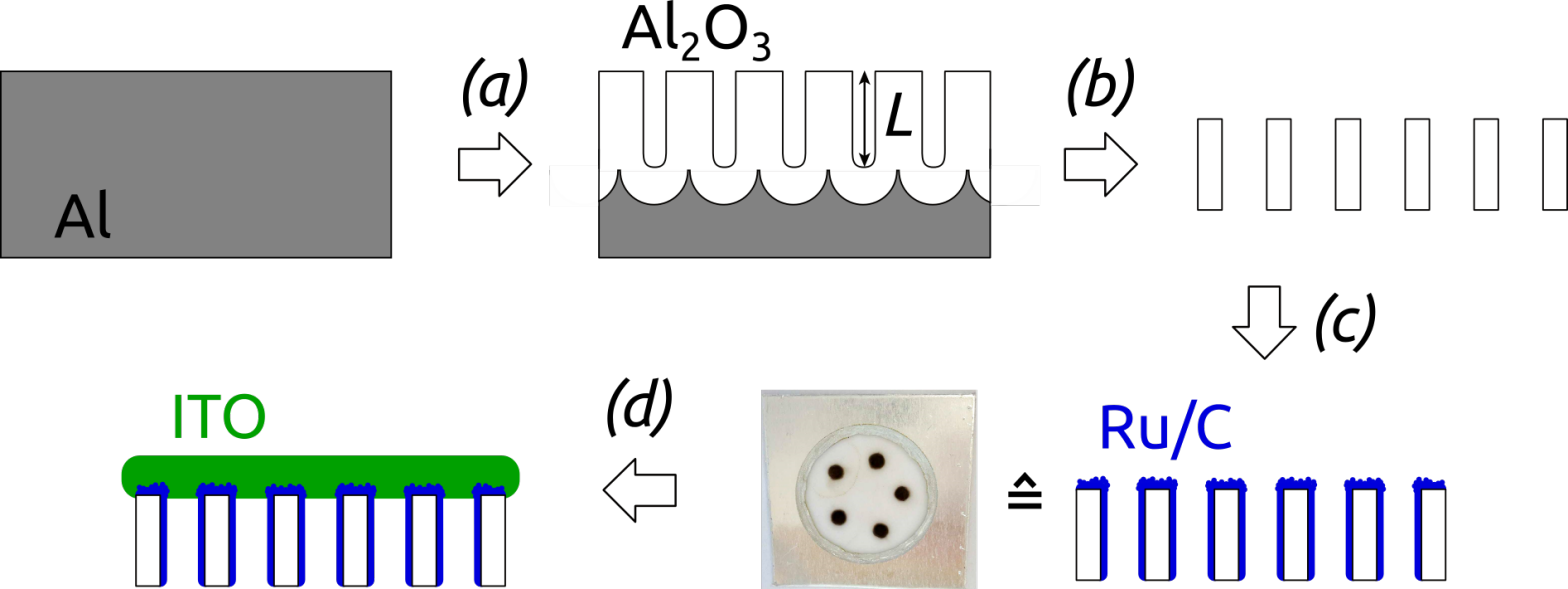
Preparation of nanostructured Ru/C electrodes. (a) Anodization of Al in 1 wt % H_3_PO_4_; this step defines the pore length *L*. (b) Removal of the Al substrate and Al_2_O_3_ barrier layer with simultaneous pore widening. (c) Laser-induced Ru/C decoration of the nanoporous Al_2_O_3_ surface. The photograph of the nanostructured surface after laser coating exhibits five well-defined dark deposition areas of 2 mm diameter on an anodic alumina substrate of 16 mm diameter. (d) Sputter-coating of the ITO backside contact.

This successful Ru/C laser deposition can now be transferred to anodic alumina templates featuring ordered arrays of parallel and cylindrical nanopores. The full preparation procedure is delineated in Figure 5. In the anodization conditions used here, the pitch *P* and diameter *D* are set to approx. 425 nm and 370 nm, respectively. This value of *D* is the maximum possible given a set *P*. It maximizes the specific surface area and thereby the electrocatalytic current density reachable [43]. The pore length is varied in the range of 11 ≤ *L* ≤ 24 μm. In contrast to planar substrates, the laser beam must be directed to the solution/substrate interface after traversing the solution (Figure 3c,d).

**Figure 6:**
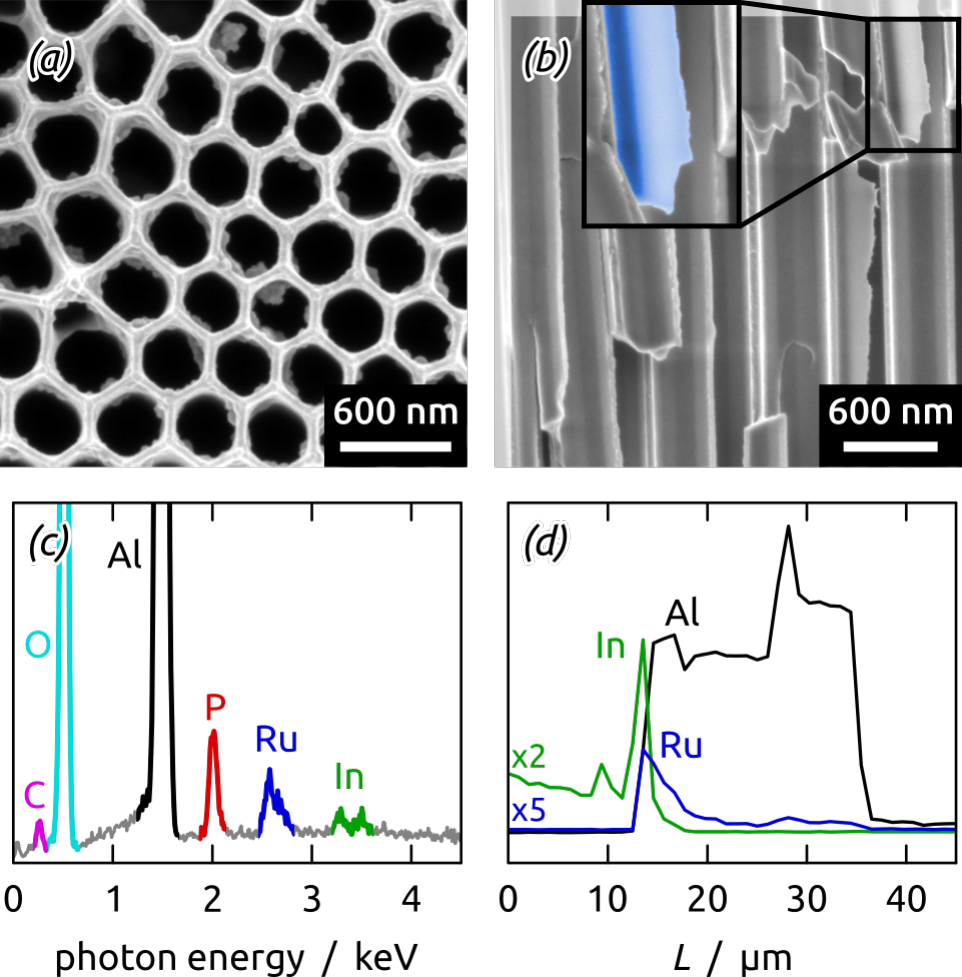
Scanning electron micrographs of a nanostructured Ru/C sample after all preparation steps in top view (a) and cross-section (b). EDX spectrum recorded over the whole sample length *L* = 24 μm (c). EDX profile taken along the cross-section (d). Laser-irradiation of Ru_3_(CO)_12_ solutions results in thin ruthenium containing coatings along the whole length of Al_2_O_3_ pores.

In these conditions, deposition durations of 15 min are sufficient for the successful deposition of thin Ru/C coatings inside the Al_2_O_3_ pores (with additional material on the front sample side, Figure 6a,d). In a last step, an ITO electrical contact is sputter-coated onto the front side of the sample. EDX spectroscopy reveals the presence of expected elements Al, O, P and In of the substrate and electrical contact, as well as Ru and C in the deposited layer (Figure 6c,d, Figure S2, Supporting Information File 1). The atomic ratios Ru/Al = 0.009 (±0.005) and C/Al = 0.137 (±0.031) (Table S1, Supporting Information File 1) demonstrate the low noble metal loading. These numbers can be expressed as 1.5 wt % Ru in our samples, or equivalently, 41 mg cm^−3^ (given the density of the Al_2_O_3_ framework) [44], or alternatively, as areal loadings, for example 53 µg cm^−2^ for a pore length of 13 µm. The volumetric value is comparable to state-of-the-art catalytic water oxidation systems based on supported RuO*_x_* (40–50 mg cm^−3^ have been reported on siliceous supports) [23–24]. The areal loadings are as low as the lowest values found in the literature (amorphous RuO_2_ with 49 µg cm^−2^ [45], mixed Ru–Pt catalyst with 15–35 µg cm^−2^ [20], and RuO_2_ nanoparticles with 49 µg cm^−2^) [46].

### Chemical characterization of nanostructured Ru/C samples

The chemical and phase identity of the Ru/C material obtained by laser-induced deposition is delivered by a combination of X-ray diffraction, Raman spectroscopy and X-ray photoelectron spectroscopy. Firstly, the Ru/C layer is amorphous, since only crystalline Al peaks of the substrate are visible in the X-ray diffraction pattern (Figure S3, Supporting Information File 1).

**Figure 7:**
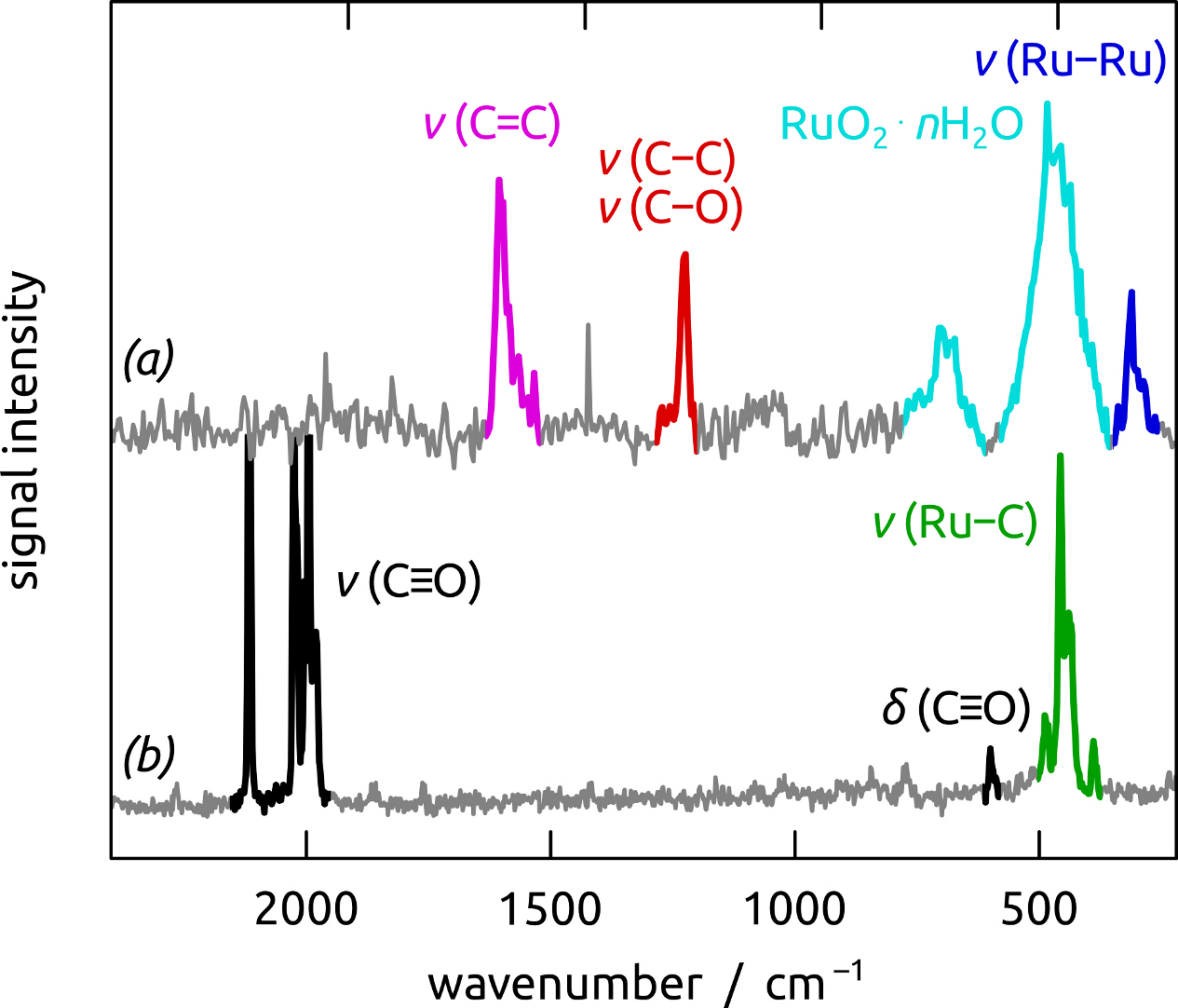
Raman spectra of a nanostructured template coated with Ru/C films, without ITO contact (a) and of the solid precursor Ru_3_(CO)_12_ (b).

The Raman spectra recorded on the Ru/C coated nanostructured sample (Figure 7a) can be divided into two distinct regions below 800 cm^−1^ and beyond it. In the low- frequency region, the broad peaks centered at 465 and 690 cm^−1^ can be attributed to hydrous ruthenium oxide (RuO_2_∙*n*H_2_O) [47–51], whereby an overlap with Ru–C stretching modes cannot be excluded (see also the signal generated by the molecular precursor, Figure 7b). The 313 cm^−1^ peak originates from metallic Ru [52–55]. In the high-frequency region, the conspicuous maximum at ≈1600 cm^−1^ is due the stretching vibration of C=C bonds in aromatic or graphitic carbon. The peak at 1224 cm^−1^ corresponds to stretching vibrations of C–C and C–O single bonds (the ‘disorder’ peak usually found for graphitic material) [56–61]. Importantly, the absence of carbonyl stretching vibrations around 1950–2190 cm^−1^ rules out any remnants of molecular precursor Ru_3_(CO)_12_ (Figure 7b [52,62]).

**Figure 8:**
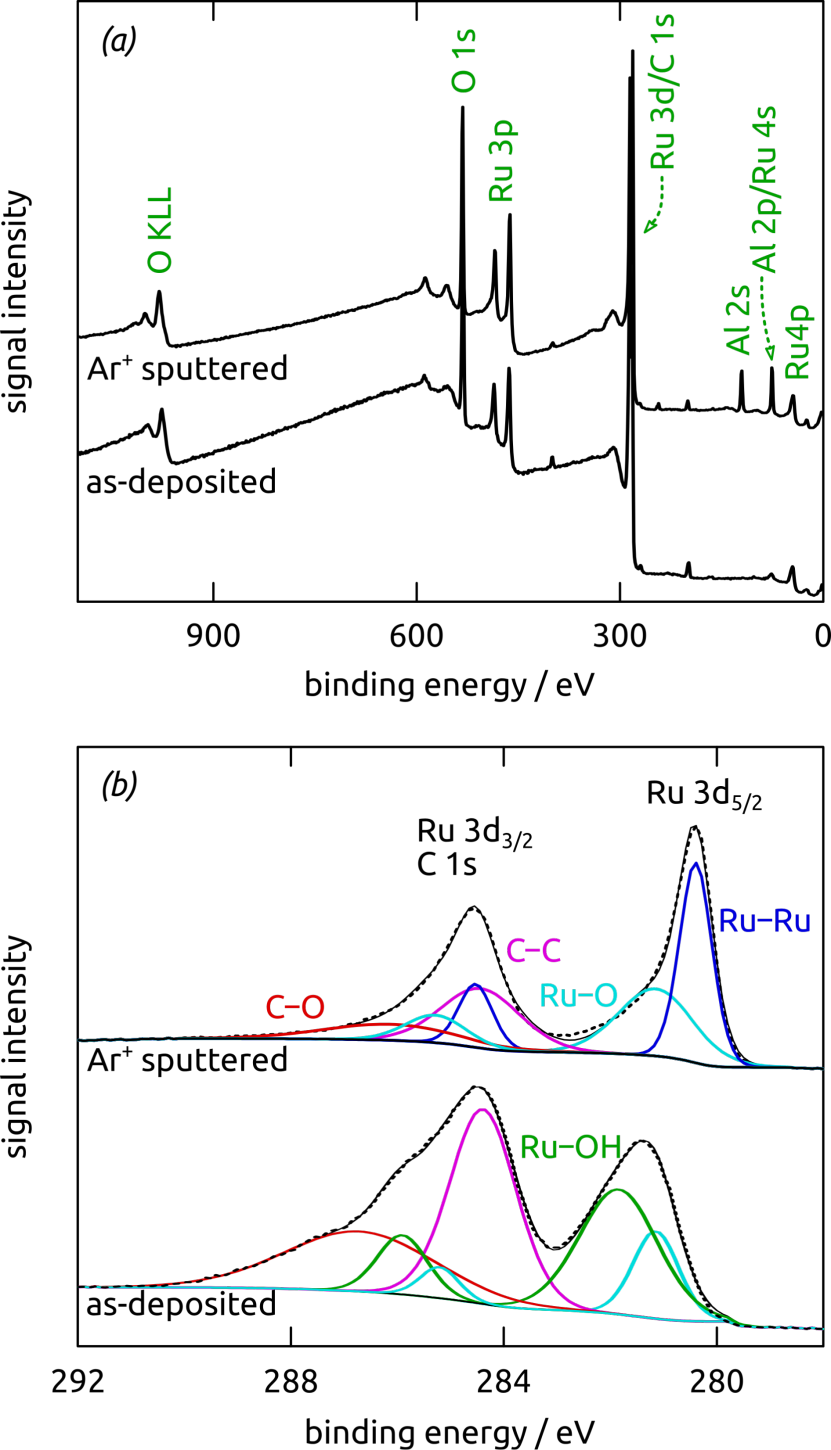
X-ray photoelectron spectra of a nanostructured Ru/C sample recorded as deposited and after Ar^+^ sputtering. All spectra are shifted to a C 1s binding energy position of 284.4 eV. (a) Survey spectra showing the expected elements. (b) Ru 3d region, which is superimposed with the C 1s region, displays the Ru 3d_5/2_ and Ru 3d_3/2_ peaks. The experimental data are provided as dashed lines, the fit as solid black lines, and the individual deconvoluted peaks are color-coded.

X-ray photoelectron spectroscopy (XPS, Figure 8) is used to differentiate between the oxidation states of ruthenium at its surface and in its inner (“bulk”). The overview XPS spectrum of an as-prepared nanostructured sample features only Ru, O and C (Figure 8a) from the Ru/C layer, whereas the Al_2_O_3_ substrate is completely covered and reveals no Al signal. Deconvolution of the Ru 3d region, which is superimposed with C 1s (Figure 8b), reveals two doublets for two chemically different Ru environments. Their Ru 3d_5/2_ maxima are located at 281.1 eV and 281.8 eV, respectively, consistent with Ru(IV) oxide and Ru hydroxide [63–65]. Large carbon contributions are additionally observable (partly O-bonded, Figure S4, Supporting Information File 1), which are due to the Ru/C layer and adventitious carbon. Argon ion sputtering results in a reduced carbon content (observable in both the C 1s and O 1s regions), as well as in a shift of the Ru 3d doublet of peaks to lower binding energies (Figure 8b and Figure S4, Supporting Information File 1). Thus, below the surface ruthenium is present in its metallic state (280.3 eV), whereas Ru(IV) oxide (281.1 eV) is still observable [63,66]. In conclusion, laser-induced deposition yields amorphous metallic Ru in close interaction with an amorphous carbonaceous C matrix, whereas the surface is completely oxidized, and in part hydrated.

### Water oxidation at nanostructured Ru/C electrodes

**Figure 9:**
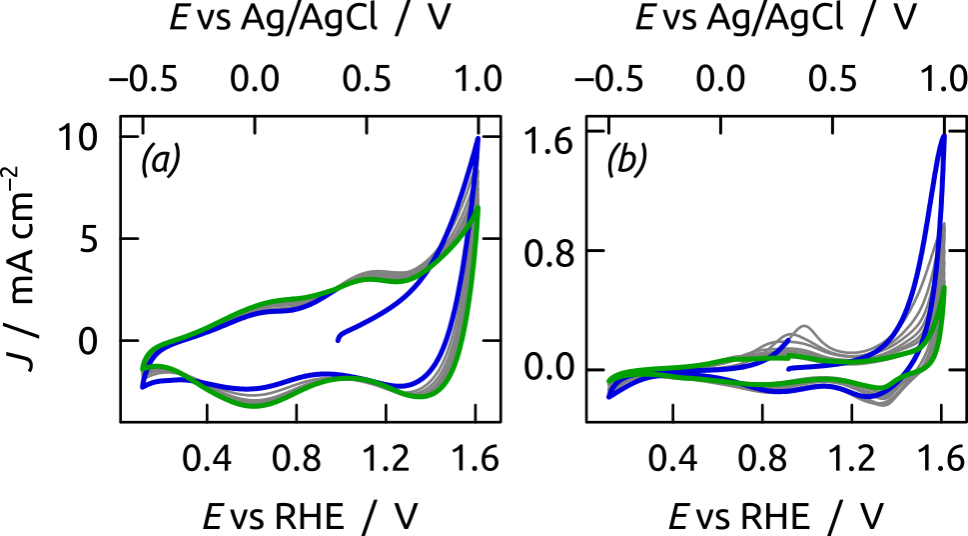
Cyclic voltammograms of Ru/C electrodes recorded in a KH_2_PO_4_ electrolyte at pH 4 (scan rate: 50 mV s^−1^). The applied potential is presented versus the reversible hydrogen electrode (RHE) and Ag/AgCl reference electrode, respectively. Comparison of 10 successive cycles for a nanostructured (*L* = 13 μm) (a) and a planar Ru/C electrode (b). The first and last cycle are highlighted in blue and green, respectively. The current densities *J* are calculated from the experimental current intensity and the macroscopically exposed sample area.

We then applied our nanoporous Ru/C electrodes to the water oxidation reaction. Therefore, an approx. 1 μm thick ITO layer at one pore extremity serves as an electrical contact. We choose pH 4 (KH_2_PO_4_ buffer) for the investigations in order to secure the stability of the Al_2_O_3_ template, ITO and ruthenium (Figure 2). In cyclic voltammetry (Figure 9a), our nanotubular nt-Ru/C electrodes feature two broad oxidative peaks located around 0.0 V and +0.5 V vs the Ag/AgCl reference electrode. These peaks correspond to the oxidation of metallic Ru to Ru(II) and subsequently to Ru(IV) at the solid surface [10,67–68]. The oxygen evolution reaction starts beyond +0.8 V (*E*’ = +0.79 V) vs Ag/AgCl at pH 4 whereas electro-corrosion to dissolved species (H_2_RuO_5_, RuO_4_^−^) begins at +0.95 V (Figure 2) [10–11]. These restrictions force us to never exceed +1.0 V applied potential. On the cathodic scan, only one reductive peak is present near 0.0 V, corresponding to the reduction of surface oxides to metallic Ru [68].

The absence of surface oxidization current during the first CV cycle (blue curve in Figure 9a) indicates that the ruthenium catalyst as prepared is present as oxidized Ru(IV). This observation is consistent with the XPS analyses presented above. The consecutive cycles yield a rather constant hysteresis area, which indicates a low loss of material in electrochemical conditions. This relative stability of our nanoporous electrode stands in stark contrast to the planar Ru/C electrode (Figure 9b).

Firstly, the current density *J* (defined with respect to the experimentally accessible macroscopic sample area) starts out 6 times lower in the planar case than for the porous electrode. Secondly, the loss of catalytic turnover of H_2_O to O_2_ visible at >0.9 V within 10 cycles is much more significant with the planar surface than with its porous counterpart. The area of the voltammetric hysteresis decreases concomitantly. These observations point to the rapid loss of noble metal catalyst from planar Ru/C surfaces.

**Figure 10:**
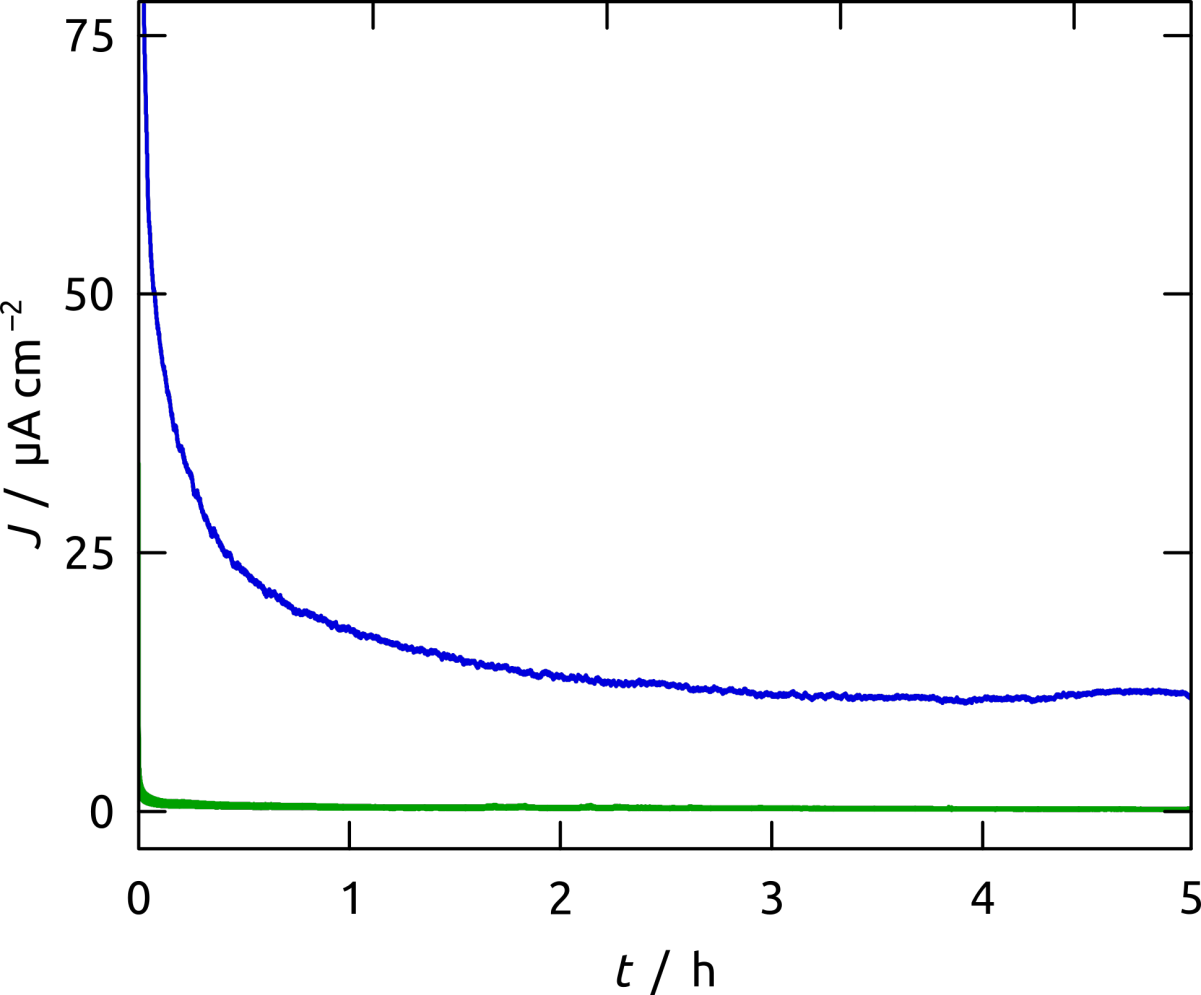
*J*–*t* curve of the same nanoporous (blue line) and planar (green line) electrode (as presented in Figure 7) during 5 h of steady-state electrolysis at +0.90 V vs Ag/AgCl.

The contrasting stability of planar and structured Ru/C electrodes can be tested further upon prolonged electrolysis. For this purpose, both types of electrodes were maintained at +0.90 V for 5 h (Figure 10). The water oxidation current density *J* on the planar Ru/C electrode declines to almost zero within about five minutes, whereas the nanostructured sample reaches a constant steady-state value *J* = 11.2 μA cm^−2^ after about three hours. This value is low, but is achieved at a very low overpotential, *η* = 0.11 V. We note that the integrated current (total charge passed over five hours) cannot be due solely to corrosion given the amount of carbon present. Thus, the application of the Ru/C laser deposition to nanoporous Al_2_O_3_ substrates yields a significant improvement of the catalyst stability in comparison to planar substrates. EDX analyses (Figures S1 and S2, Table S1, Supporting Information File 1) and cyclic voltammetry (Figure S5, Supporting Information File 1) performed after long-term bulk electrolyses support this statement.

### Optimization of electrocatalytic performance

**Figure 11:**
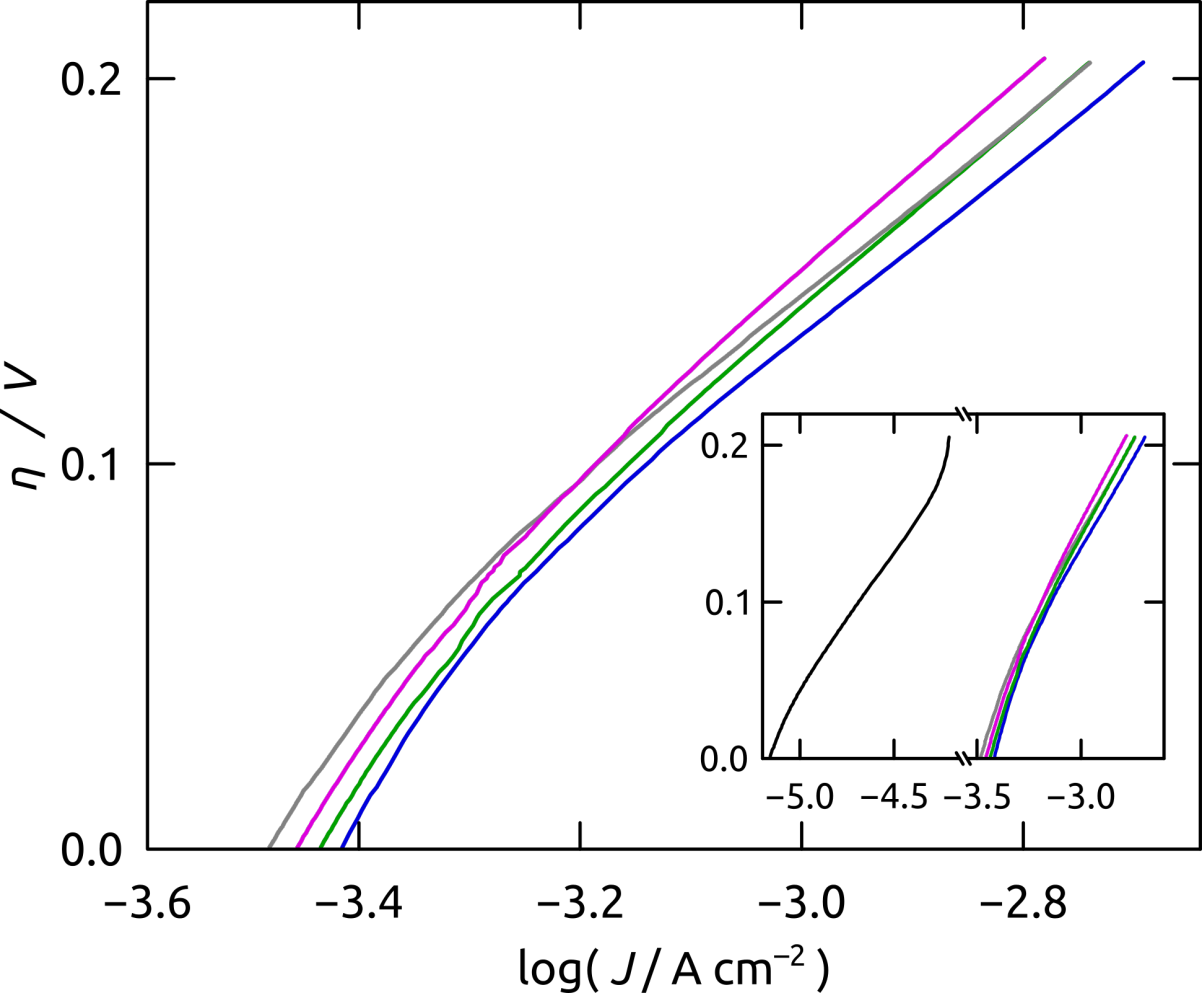
Tafel plots of nanoporous Ru/C electrodes of various lengths 11 ≤ *L* ≤ 24 μm in quasi-steady-state conditions. Tafel plots were obtained from linear sweep voltammetry (scan rate 5 mV s^−1^) measured from +0.79 ≤ *E* ≤ +1.00 V vs Ag/AgCl (corresponding overpotentials 0.00 ≤ *η* ≤ 0.21 V). Average log *J* values for a minimum of 4 samples are presented for 11 μm (gray line), 13 μm (blue line), 18 μm (green line) and 24 μm (purple line). The inset compares all nanostructured electrodes with planar Ru/C electrodes (black line).

Our preparative procedure now allows us to adjust the geometric parameters of the nanoporous substrate in order to optimize the catalytic turnover of H_2_O to O_2_. Specifically, we will study the dependency of the current density *J* on the electrodes’ geometric surface area via the variation of the pore length *L* (maintaining a constant pore diameter *D*). Figure 11 illustrates Tafel plots of Ru/C electrodes determined for pore lengths 11 ≤ *L* ≤ 24 μm measured within an applied overpotential range of 0.00 ≤ *η* ≤ 0.21 V (+0.79 ≤ *E* ≤ +1.00 V vs Ag/AgCl). All curves follow a similar trend and are located in close proximity of each other. However, the best performer is not the electrode type with the longest pores of *L* = 24 μm. Instead, those with 13 μm length yield the largest current densities at all overpotentials. They enable a water oxidation turnover that exceeds those of planar electrodes (*L* = 0 μm, Figure 11 inset) by approximately 1.5 decimal logarithmic units, or, equivalently, a factor 35.

**Figure 12:**
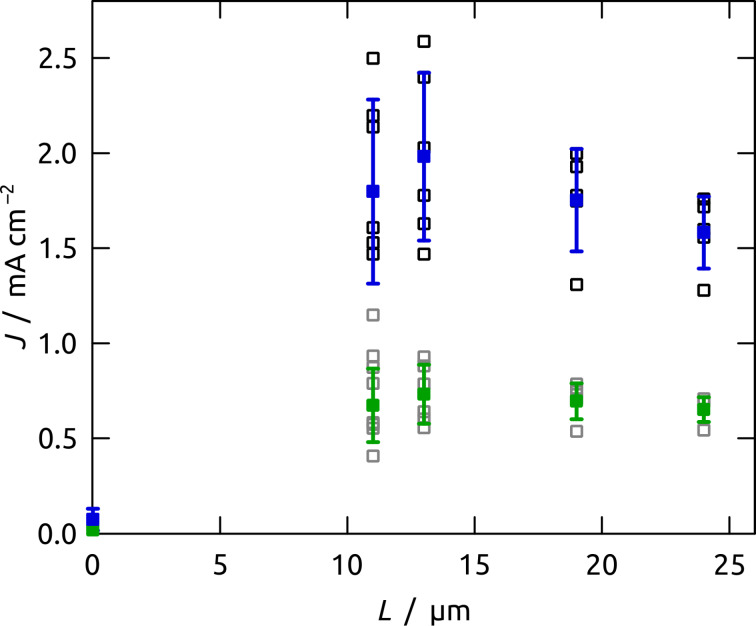
Current densities of Ru/C electrodes for water oxidation measured at pH 4 and at 0.10 V or 0.20 V applied overpotential. The current densities *J* are presented for individual samples in gray (*η* = 0.10 V) and black (*η* = 0.20 V), whereas the full green and blue squares correspond to the average values.

A clearer view of the length effect is provided by plots of *J*–*L* dependence at two distinct overpotentials, *η* = 0.10 V (mean values as green data points in Figure 12) and *η* = 0.20 V (blue data points). In both cases, pore elongation yields a rapid current density increase until a maximum is reached at 13 μm, followed by an activity loss for *L* > 13 μm. The current density loss is even more pronounced for *η* = 0.20 V than for 0.10 V, which can be attributed to transport limitation, since diffusion becomes more limiting at faster catalytic turnover. A similar observation was already made for Fe_2_O_3_-coated Al_2_O_3_ nanopores in the oxygen evolution reaction [69–71].

## Conclusion

With this, we have established a novel type of nanostructured Ru/C composite electrode for the oxygen evolution reaction at pH 4 by laser-induced deposition. Laser irradiation of Ru_3_(CO)_12_ in 1,2-dichloroethane at 325 nm provides the first laser-induced coatings of hybrid Ru/C material on planar and porous substrates. Along ordered cylindrical pores of high aspect ratio, the method yields thin and continuous films consisting of surface-oxidized metallic Ru incorporated in an amorphous carbonaceous carbon matrix.

This system applicable to the water oxidation reaction is advantageous with respect to the state of the art in several regards (Table 1) [14,18,20,28,45–46,72]:

**Table 1:** Comparison of loadings 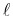, current densities *J*, and activities (specific currents *i_sp_* = *J*/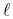) between selected literature precedents and the current results. At an overpotential of 0.2 V, our system reaches current densities comparable to the highest of the literature with Ru catalyst loadings comparable to the lowest in the literature. The activity is correspondingly maximal.

	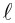 µg cm^−2^	*J_η = _*_0.2 V_ mA cm^−2^	*i_sp, η = _*_0.2 V_ A g^−1^	measurement conditions

**this work**	**53**	**2.6**	**49.1**	**5 mV s^−1^**
Shao-Horn [46]^a^	50	0.01	0.2	10 mV s^−1^
Kokoh [14]	380	0.8	2.1	5 mV s^−1^
Haverkamp [18]	160	1.0	6.3	20 mV s^−1^
Mayrhofer [72]	370	5.0	13.5	10 mV s^−1^
Strasser [20]	(14–35)	0.8	(23–56)	5 mV s^−1^
Scott [28]	2000	232	116	/
Nakato [45]	49	7.5	154	5 mV s^−1^

^a^The values reported in the Shao-Horn paper [46] are without internal resistance correction (to allow for a relevant comparison with other papers), at a slightly more elevated overpotential of *η* = 0.25 V.

1. Moderate current densities can be achieved at very low overpotential upon optimization of the nanoporous geometry. For example, pores of *L* = 13 μm deliver 2.6 mA cm^−2^ at *η* = 0.20 V. This value not only represents a 35-fold increase with respect to planar electrodes, it also is competitive with state-of-the-art ruthenium-based water oxidation electrodes (Table 1).
2. The nanoporous geometry, combined with the embedding of noble metal inside the carbonaceous matrix, stabilizes the electrocatalyst to the point that a steady state is reached upon continuous electrolysis. The current then stays stable for several hours. The comparison with the literature shows that only Kokoh et al. tested the long-term stability of their Ru-based electrocatalyst [14].
3. The fine dispersion of Ru in the conductive matrix and the high degree of geometric control afforded by the alumina support complement each other to optimize the contact between catalyst and electrolytic solution while minimizing transport effects. The noble metal loading can thereby be reduced to 53 µg cm^−2^, and its activity optimized to 49 A g^−1^ (for *L* = 13 μm and *η* = 0.20 V), which is comparable to the best literature values (see Table 1 for a systematic comparison).
4. The metal/carbon composite is obtained in a single deposition step from a commercially available compound in an experimentally simple procedure.

The improved stability demonstrated here, the significant current densities, and the large activities obtained at low overpotential question the relative neglect of ruthenium in the water oxidation literature. This less costly metal could, given proper preparation procedures, advantageously replace iridium for some applications – most prominently electrical energy storage under nearly reversible conditions.

## Experimental

### Materials

Chemicals were purchased from Sigma-Aldrich, VWR, or Roth and used as received. Water was purified in a Millipore Direct-Q system for the application in electrolytes. As planar substrates, microscope cover glasses (borosilicate glass, 18 × 18 mm^2^, 0.13–0.16 mm thickness) were purchased from Marienfeld-Superior. Aluminum plates (99.99%) for the anodization procedure were supplied by Smart-Membranes. The indium tin oxide sputter target (99.99%) was purchased from AEM.

### Preparation of planar samples

For planar electrodes microscope cover glasses were ultrasonically cleaned with ethanol and water, then dried in a flow of nitrogen. In a next step, the slides were sputter-coated with approx. 700 nm of indium tin oxide (ITO) in radio frequency (RF) mode in a reactor from Torr International Inc. The conductive layer was subsequently annealed in N_2_ atmosphere for 4 h at 400 °C in a high-temperature P330 furnace from Nabertherm. The planar substrates were then coated with Ru/C layers via laser-induced deposition. The precursor solution was prepared by ultrasonic dissolving of 1 mg of triruthenium dodecacarbonyl (Ru_3_(CO)_12_) in 1 mL of 1,2-dichloroethane (C_2_H_4_Cl_2_) of analytical grade purity for 15–20 min. To remove the undissolved components, the solution was centrifuged with a SIGMA 2-16P centrifuge at 10000 rpm for 5 min. A microcuvette (*d* = 9 mm) was filled with the precursor solution and covered with the planar substrate with the ITO side facing the solution. Bubble formation in the solution was prevented via axial sliding of the substrate on the cuvette. An unfocused beam (ca. 2 mm in diameter) of a Plasma He-Cd laser (continuous wave (CW), λ = 325 nm, *P* = 10 mW) was directed to the substrate/solution interface from the side of glass for an irradiation duration of 30 min (Figure 3a,b). The maximum temperature in the laser focal spot does not exceed 27 °C as recorded with a Thermovision camera Ti32 from Fluke. After the deposition procedure the planar samples were washed in a flow of acetone and isopropanol.

### Preparation of nanostructured samples

Nanostructured Ru/C electrodes were prepared in several steps as illustrated in Figure 5. A standard two-step anodization of aluminum (represented as (a) in Figure 5) delivered the nanoporous aluminum oxide membranes further used as templates [35]. Aluminum plates of 2.2 cm diameter were anodized in home-made two-electrode cells consisting of a PVC beaker with four circular openings at the bottom. They were therefore held between an O-ring and a thick copper plate operating as an electrical contact. Adequate cooling of the beaker was ensured via a cold plate connected to a closed-circuit cooler by Haake. The PVC beaker was filled with the electrolyte and closed with a lid equipped with a mechanical stirrer and silver wire mesh as counter-electrode. The whole setup was thermally insulated laterally. Electropolishing of the aluminum plates in a cooled perchloric acid/ethanol solution (1:3 v/v HClO_4_/EtOH) for 5 min under +20 V represented the first process step. They were then rinsed, cooled and anodized under +195 V for 23 h at 0 °C in 1 wt % H_3_PO_4_. In the following, the anodized plates were exposed to a chromic acid solution (0.18 M CrO_3_ in 6 wt % H_3_PO_4_) for 23 h at 45 °C for the removal of the disordered, porous Al_2_O_3_ generated. The second anodization was performed subsequently for 3, 4, 6 or 8 h at 0 °C in 1 wt % H_3_PO_4_ in order to vary the pore length. The next procedures (step (b) of Figure 5) included removing the metallic Al on the backside of the anodized Al_2_O_3_ with 0.7 M CuCl_2_ solution in 10% HCl, followed by opening the Al_2_O_3_ barrier layer closing the pores with simultaneous isotropic pore widening in 10 wt % H_3_PO_4_ at 45 °C for 37 to 47 min. The laser-induced deposition of Ru/C coatings followed was adapted from the planar case (vide supra) (Figure 5 step (c)). 10 μL of the 1 mg/mL Ru_3_(CO)_12_ dichloroethane solution were dropped on the Al_2_O_3_ templates. The laser beam was directed to the solution/substrate interface from the side of the solution droplet (see Figure 3c,d) with an irradiation time of 15 min. As soon as the solution evaporated (approx. each 30 s) a new droplet was placed on the same spot. The nanostructured samples were then washed in a flow of acetone and isopropanol. Four to five depositions were performed on each substrate. In a last step, the electrical contact was generated by sputter-coating of approx. 1 μm ITO in RF mode on the sample side of laser deposition (step (d) in Figure 5).

### Instrumental methods

Scanning electron micrographs were obtained on a Zeiss Merlin field-emission SEM with a field-emission cathode and standard In-lens SE and SE2 detectors. All measurements were performed in the chamber with a base pressure in the range of 10^−7^ mbar. The acceleration voltage was 10 to 1 keV with a beam current of 124–450 pA. Line averaging procedure was used for all images to reduce noise. Energy dispersive X-ray spectroscopy (EDX) was obtained on a JEOL JSM 6400 PC implemented with a LaB_6_ cathode and silicon drift detector (SDD). All Raman spectra were collected at room temperature in a backscattering geometry using a Horiba Jobin-Yvon LabRam HR 800 Raman spectrometer equipped with an Olympus BX41 microscope. The spectra were obtained with 488 nm radiation from an Ar^+^ gas laser and recorded in the 35–3290 cm^−1^ spectral range. The acquisition time was set to 500 s. The laser power was focused with a 100× objective on the sample and always kept at 5.4 μW. The spectra presented in this work are averaged from at least 5 measurements. The spectra were processed with LabSpec 5.78 including spike removal and baseline correction. The crystal structure was studied by powder X-ray diffraction measurements using a Bruker D8 Advance diffractometer in reflection mode and with Cu K_α1_ radiation (λ = 1.54056 Å) and LynxEye XE-T detector. Monochromatized Al K_α_ XPS spectra were acquired on a PHI Quantera II system with a base pressure of 10^−9^ mbar. Adventitious carbon was removed from the surface by 1 min, 2 kV Ar^+^ ion sputtering. To prevent charging a combination of electron and ion neutralization was employed. The Ru 3d and O 1s XPS core level spectra were analyzed using a fitting routine which decomposes each spectrum into individual mixed Gaussian–Lorentzian peaks using a Shirley background subtraction over the energy range of the fit. Finally, all spectra were shifted to give a C 1s binding energy position of 284.4 eV to correct for a slight overcompensation in the neutralization.

### Electrochemical studies

Planar samples were laser-cut with a GCC LaserPro Spirit LS Laser into smaller areas of 1 cm^2^, placed on small copper plates, whereby the electrical contact was established by double-sided conductive copper foil at the edges of the glass slide. In the case of nanostructured samples the individual deposition areas were laser-cut and subsequently glued with the ITO contact on small copper plates using double-sided conductive copper foil. A chemically resistant and electrically insulating polyimide (Kapton^®^) adhesive tape featuring a laser-cut circular window of 1.5 mm diameter was used to define the sample area exposed to the electrolyte. This macroscopically defined exposed sample area of 0.018 cm^2^ is the value *A* used to define current densities (*J* = *I*/*A)* from the measured currents *I*. The samples were then adjusted into three-electrode electrochemical cells, exposing the defined sample area to a pH 4 phosphate electrolyte prepared from 0.1 M KH_2_PO_4_. The stability of the Al_2_O_3_ template and ITO backside contact in pH 4 conditions was verified with SEM after 20 h in the electrolyte. All electrochemical measurements including cyclic voltammetry (CV), linear sweep voltammetry (LSV) and steady-state electrolysis were performed from the open-circuit potential at room temperature using Gamry Interface 1000 potentiostats. The standard redox potential of the Ag/AgCl/KCl(sat.) reference electrode is shifted by +0.20 V relative to the normal hydrogen electrode (NHE). Cyclic and linear sweep voltammograms were measured at scan rates of 50 mV s^−1^ or 5 mV s^−1^, respectively. Using the LSV data, Tafel plots were obtained for +0.79 V ≤ *E* ≤ +1.00 V vs Ag/AgCl (overpotentials 0.00 ≤ *η* ≤ 0.21 V). Steady-state electrolysis was measured for 5 h at +0.90 V. Additionally, a control experiment performed with a pure ITO contact on flat and nanostructured substrates demonstrated that the presence of ITO is irrelevant to the electrochemical performance.

## Supporting Information

File 1 Additional experimental data.
