# Supplementary material for: Nanoporous water oxidation electrodes with a low loading of laser-deposited Ru/C exhibit enhanced corrosion stability
Source: Beilstein J Nanotechnol. 2019 Jan 11;10:157–67. doi: 10.3762/bjnano.10.15 (PMC6334789; doi:10.3762/bjnano.10.15)
Supplement: File 1 — Additional experimental data. [file Beilstein_J_Nanotechnol-10-157-s001.pdf]

BEILSTEIN JOURNAL OF NANOTECHNOLOGY

Supporting Information

for

Nanoporous water oxidation electrodes with a low loading of laser-deposited Ru/C exhibit enhanced corrosion stability

Sandra Haschke, Dmitrii Pankin, Vladimir Mikhailovskii, Maïssa K. S. Barr, Adriana Both-Engel, Alina Manshina and Julien Bachmann

Beilstein J. Nanotechnol. **2019,** *10,* 157–167. doi:10.3762/bjnano.10.15

Additional experimental data

License and Terms: This is a supporting information file under the terms of the Creative Commons Attribution License (http://creativecommons.org/licenses/by/4.0). Please note that the reuse, redistribution and reproduction in particular requires that the authors and source are credited.
The license is subject to the *Beilstein Journal of Nanotechnology* terms and conditions: (https://www.beilstein-journals.org/bjnano)

Contents:

Energy-dispersive X-ray analyses of planar Ru/C electrodes, Figure S1 S2

Energy-dispersive X-ray analyses of nanoporous Ru/C electrodes, Figure S2, Table S1 S3

X-ray diffraction pattern of nanoporous Ru/C samples, Figure S3 S4

X-ray photoelectron spectroscopy of nanoporous Ru/C samples, Figure S4 S5

Cyclic voltammetry of planar and nanoporous Ru/C electrodes, Figure S5 S6

References S6

Energy-dispersive X-ray analyses of planar Ru/C electrodes

The presence of ruthenium and carbon in the laser-induced coating on planar substrates is proven by EDX spectra of the pure and coated substrate. One can observe the expected elements Si, O, In, Sn and N attributable to the planar ITO coated substrate. The Ru/C sample reveals a distinct Ru signal, as well as a pronounced C peak. After electrochemical measurements, however, the Ru peak has vanished, indicating a loss of the catalyst layer.

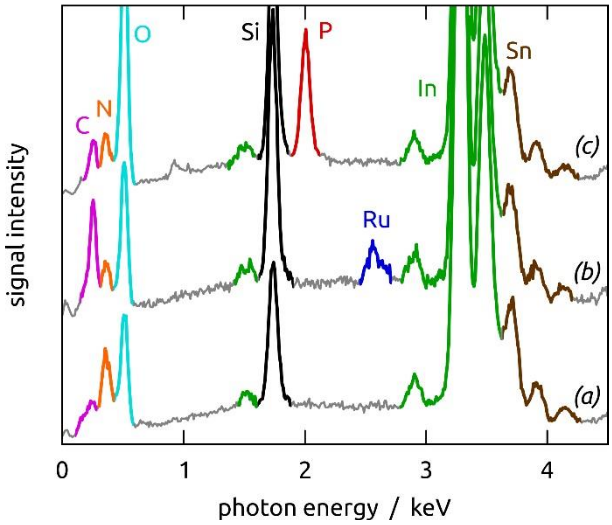

Figure S1: EDX spectra recorded on an ITO coated microscope cover glass *(a)*, an as-prepared Ru/C coated planar sample *(b)* and the same sample after electrochemical measurements *(c)*.

Energy-dispersive X-ray analyses of nanoporous Ru/C electrodes

The successful deposition of Ru/C coatings on nanostructured substrates is shown by EDX analyses. In contrast to the pure anodic alumina template, distinct ruthenium and carbon peaks are present after laser deposition besides the expected substrate elements Al, P and O. Furthermore, the Ru/C thin film reveals significant stability upon electrochemical treatment since both elements are still detectable after electrolysis.

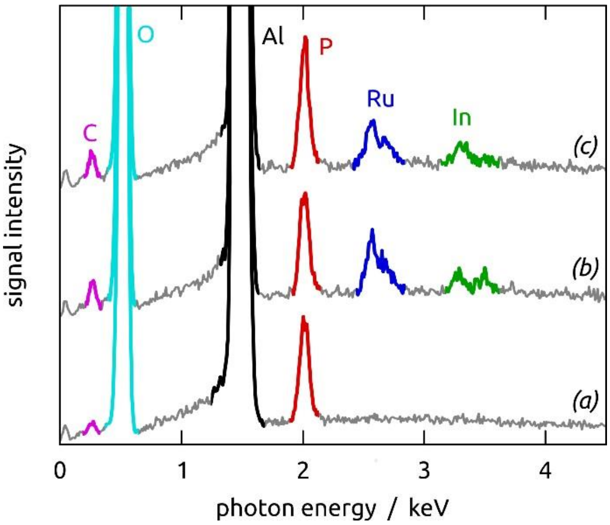

Figure S2: EDX spectra recorded on an alumina template *(a)*, an as-prepared Ru/C coated nanostructured sample *(b)* and the same sample after electrochemical measurements *(c)*. Both samples are equipped with an ITO backside contact, L = 24 µm.

Table S1: Average ruthenium and carbon loadings (related to aluminum in the matrix) for nanostructured Ru/C samples calculated from EDX analyses (averages of values obtained for several distinct samples). Comparison is drawn between a pure alumina template, as-deposited nanostructured Ru/C samples, and the same samples after electrochemical treatment.

sample	*Ru/Al atomic ratio*	*C/Al atomic ratio*
pure Al_2O_3	0.0	0.019
as-deposited Ru/C-coated Al_2O_3	0.009 (±0.005)	0.033 (±0.003)
electrochemically treated Ru/C coated Al_2O_3	0.005 (±0.002)	0.030 (±0.004)

X-ray diffraction pattern of nanoporous Ru/C samples

Laser-induced deposition yields amorphous Ru/C thin films on anodic alumina substrates since only crystalline Al peaks of the aluminum substrate framework are observable in the X-ray diffraction pattern.

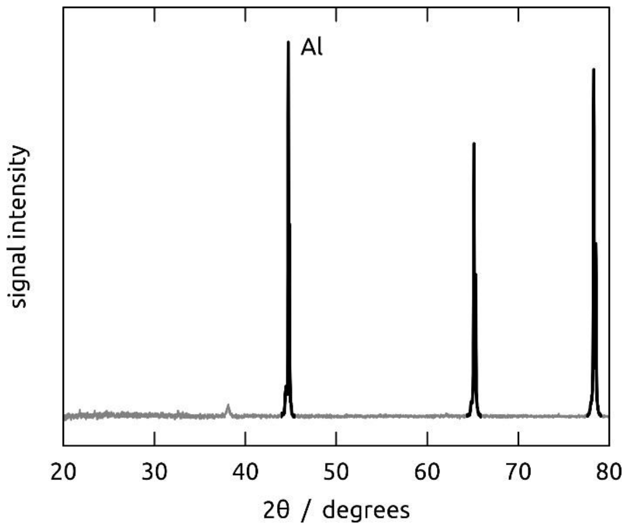

Figure S3: X-ray diffraction pattern of a nanostructured Ru/C sample. The aluminum peaks are referenced to COD 9008460, L = 24 µm.

X-ray photoelectron spectroscopy of nanoporous Ru/C samples

The as-deposited nanostructured Ru/C sample exhibits large amounts of oxidized carbon as well as hydroxyl/H_2O contributions in the O 1s region [1,2]. After Ar^+ sputtering, however, deconvolution reveals only one peak, which corresponds to oxygen bound to aluminum in the Al_2O_3 template [3]. This observation is in line with deconvolution of the Ru 3d and C 1s region, showing an oxidized Ru/C surface after sample exposure to air. After subsequent removal of the upper catalyst layer, however, ruthenium in the metallic state and aluminum plus Al_2O_3 of the substrate are detectable.

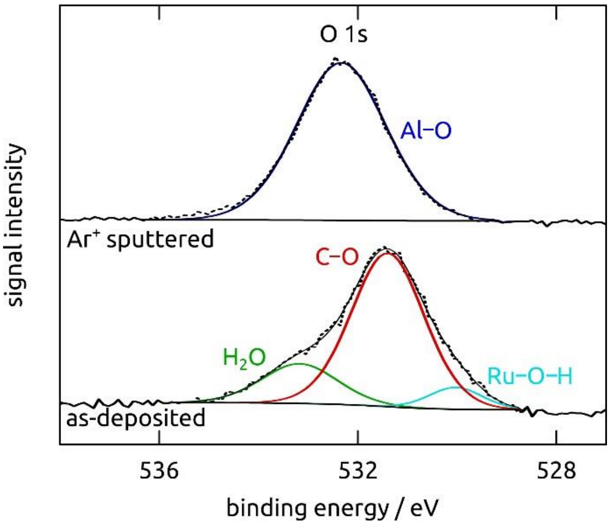

Figure S4: Chemical composition of oxygen (O 1s) from X-ray photoelectron spectroscopy recorded on a nanostructured Ru/C sample as-deposited and after Ar^+ sputtering. All spectra are shifted to a C 1s binding energy position of 284.4 eV, L = 24 µm.

Cyclic voltammetry of planar and nanoporous Ru/C samples

Freshly prepared planar and nanostructured Ru/C electrodes both not only exhibit typical features attributable to ruthenium/ruthenium oxide surfaces [4-6], but also to the oxidation of water at $E \geq +0.79$ V. After quasi-steady-state measurements, the Ru/C catalyst thin film has probably already detached from the planar electrodes, whereas nanostructured electrodes still reveal similar CV shapes with somewhat reduced intensity. Additionally, nanotubular Ru/C electrodes show improved stability upon long-term steady-state electrolysis. Thus, the use of nanoporous anodic alumina as a template provides a significant advantage compared to planar glass substrates.

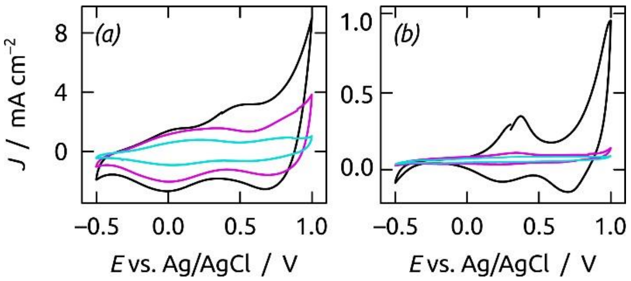

Figure S5: Cyclic voltammograms of a nanostructured *(a)* and planar *(b)* Ru/C sample comparing their stability upon electrochemical treatment. The CVs were recorded in 0.1 KH_2PO_4 at pH 4 vs an Ag/AgCl electrode, whereas the second of 10 successive cycles is presented here. Black: as-deposited, pink: after linear sweep voltammetry, blue: after 5 hr of steady-state electrolysis at +0.90 V, L = 13 µm.

References

1. Morgan, D. J. *Surf. Interface Anal.* **2015**, *47*, 1072-1079.
2. Wang, W.; Zhong, J.; Favors, Z.; Ozkan, C. S.; Guo, S.; Lee, I.; Zaera, F.; Ahmed, K.; Ozkan, M. *Sci. Rep.* **2014**, *4*, 4452.
3. Treverton, J. A.; West, R.; Johnson, D.; Thornton, M. *Appl. Surf. Sci.* **1993**, *72*, 349-361.
4. Burke, L. D.; Mulcahy, J. K. *J. Electroanal. Chem.* **1976**, *73*, 207-218.
5. Lezna, R. O.; De Tacconi, N. R.; Arvía, A. J. *J. Electroanal. Chem.* **1983**, *151*, 193-207.
6. Galizzioli, D.; Tantardini, F.; Trasatti, S. *J. Appl. Electrochem.* **1974**, *4*, 57-67.
